# Burnout and Quality of Work Life among Physicians during Internships in Public Hospitals in Thailand

**DOI:** 10.3390/bs14050361

**Published:** 2024-04-25

**Authors:** Vithawat Surawattanasakul, Wuttipat Kiratipaisarl, Penprapa Siviroj

**Affiliations:** 1 Department of Community Medicine, Faculty of Medicine, Chiang Mai University, Chiang Mai 50200, Thailand; vithawat.surawat@cmu.ac.th (V.S.); wuttipat.k@cmu.ac.th (W.K.); 2 Environmental and Occupational Medicine Excellence Center, Faculty of Medicine, Chiang Mai University, Chiang Mai 50200, Thailand

**Keywords:** quality of work life, burnout, intern physicians, public hospitals, Thailand

## Abstract

Physicians are exposed to occupational stress and burnout, which have been identified as contributing to a decrease in the quality of work life (QWL). Thailand’s medical education program, consisting of a six-year curriculum with government tuition support followed by three years of internships, provides the context for this investigation. This study aimed to assess the QWL among intern physicians (IPs) in public hospitals and investigated the association between burnout and QWL. A cross-sectional study was conducted among 241 IPs in public hospitals in Thailand utilizing an online self-administered questionnaire. The questionnaire included a Thai version of a 25-item QWL scale and the Maslach Burnout Inventory—Human Services Survey for Medical Personnel. Data analysis was performed using multivariable logistic regression. A significant proportion of IPs experienced low to moderate QWL (72.6%), with low levels of home–work interface (39.4%) and employee engagement (38.6%). In the exploratory model, after adjusting for sex and age, IPs with high depersonalization and low personal accomplishment demonstrated an association with low QWL (adjusted OR, aOR 2.08, 95% CI 1.01 to 4.31; aOR 2.74, 95% CI 1.40 to 5.39). Healthcare organizations should regularly assess intern physicians’ QWL and burnout, prioritizing interventions; ensure reasonable work hours, schedule adjustments, and open communication; and develop support systems for cost-effective interventions. Further research on the dynamic relationship between burnout and QWL is crucial for targeted and culturally sensitive interventions.

## 1. Introduction

Quality of work life (QWL) specifically focuses on the aspects of an individual’s experience related to their work environment and conditions, encompassing employee satisfaction, work–life balance, stress levels, job security, and career achievements [1]. In recent years, the QWL within the healthcare sector, particularly in hospital settings, has been a critical concern due to its multifaceted impact on employees and patient care [2,3]. The intricate nature of the healthcare environment, coupled with the demanding responsibilities and challenges faced by medical professionals, underscores the importance of addressing factors contributing to a low QWL. High levels of QWL attract and motivate employees and contribute to good work performance, cultivating an atmosphere of trust and mutual respect within the organization [4,5]. Conversely, a low QWL can have detrimental effects on both physical and mental health, as well as lead to poor work performance or maladjusted behavior, such as errors and substandard patient care [3,6,7].

Physicians, in particular, face unique stressors stemming from their work patterns, including irregular hours and the prioritization of patient needs over their well-being. The prevalence of work-related stress among physicians is strongly associated with various factors, such as role conflict, emotional demands, concerns about medical errors, and exposure to verbal or physical aggression [8,9]. Additionally, demanding schedules and interpersonal conflicts further exacerbate these stressors, leading to adverse effects on their mental health, job satisfaction, and retention within the profession [10,11,12,13].

Burnout and QWL are interconnected concepts, especially within the healthcare sector, where burnout often serves as a significant indicator of a poor QWL [14,15,16,17]. Maslash, defining burnout as assessed by the Maslach Burnout Inventory—Human Services Survey for Medical Personnel, MBI-HSS (MP), described it as a complex syndrome characterized by emotional exhaustion (EE), depersonalization (DP), and reduced personal accomplishment (PA) [18]. It is related to chronic workplace stressors, such as heavy workloads, long work hours, and the emotional strain of making critical decisions [19,20,21]. On the other hand, QWL encompasses a wide range of factors beyond workloads and hours, including job satisfaction, work–life balance, organizational culture, growth opportunities, autonomy, and social support [21]. The Work-related Quality of Life Scale (WRQLS), initially developed in England by Van Laar et al., comprises 23 items designed to assess QWL across six dimensions: job and career satisfaction (JCS), general well-being (GWB), home–work interface (HWI), stress at work (SAW), control at work (CAW), and working conditions (WCS) [22,23]. Translated into 13 languages, including Thai, this scale has been widely used across various organizational settings [24]. In Thailand, a brief Thai version of the Work-related Quality of Life Scale-2 Online Website (brief THWRQLS) was developed from the WRQLS by Chaiear. This tool offers a practical means of assessing QWL among physicians [23,25].

Thailand’s health system is primarily hospital-based, with public hospitals holding the majority of hospital beds. In Thailand, the medical education system provides a six-year study period for high school diploma holders, with government-funded tuition support at public institutions. Upon graduation, scholarship recipients are required to serve three years, including a dedicated internship year in provincial hospitals. During the internship, the first year entails rotation through various wards, such as internal medicine, surgery, and orthopedics, as well as elective subjects, including pediatrics, obstetrics, gynecology, emergency, and community hospitals. This structured program offers a comprehensive twelve-month training experience [26,27,28]. The successful completion of this first-year internship is a prerequisite for further advancement into any medical resident training program [29]. However, physicians in public hospitals face notably high workloads, largely due to Thailand’s policy of universal coverage. In 2023, the average number of outpatients visited in public hospitals reached three times per person per year [30]. Despite the demanding, mandatory, and often unpredictable nature of their working conditions, the QWL of interns in these settings has received limited attention. Consequently, Thai physicians working in public hospitals often experience high levels of stress and reduced well-being.

Previous studies in Thailand employing the same instrument to assess QWL as the brief THWRQLS used in this study have revealed concerning findings [31,32,33]. For example, 67.5% of physicians in public hospitals in Thailand were reported to have a low QWL. Similarly, 61.6% of physicians in a university hospital were found to have a low to moderate QWL, while 76.6% of medical residents reported experiencing a moderate QWL [31,32,33]. Additionally, burnout is prevalent among residents in Thailand, with reported rates ranging from 17% to 53% across various specialties [34,35,36,37,38,39]. Moreover, physicians experiencing burnout often encounter various challenges, such as poor well-being, poor work–life balance, a lack of resources and support, career disengagement, decreased satisfaction, and increased psychological demands [15,40,41,42,43]. Previous international studies exploring the potential impacts of physician burnout on healthcare efficiency have largely neglected to consider its association with physician career engagement. Additionally, these studies indicate an association between healthcare provider burnout and a decline in the quality of patient care [43,44,45].

However, to our knowledge, no published studies have investigated the association between burnout and QWL among physicians working in public hospitals in Thailand, particularly those in internship positions. The existing literature has primarily focused on burnout or QWL and its determinants among general physicians and medical residents in public hospitals. In this study, we utilized the MBI-HSS (MP) translated into the Thai version [46] and the brief THWRQLS [25]. This QWL tool encompasses seven dimensions relevant to the work environment, making it suitable for assessing QWL among physicians undergoing medical training. This study aimed to assess the QWL among intern physicians working in public hospitals in Thailand, with a specific focus on investigating the association between burnout subscales and their QWL.

## 2. Materials and Methods

### 2.1. Study Design and Participants

This cross-sectional study was conducted online from June to July 2022 among first-year intern physicians working in Thailand. A web-based online questionnaire was used in the study during the last two months of the academic year’s internship. Our participants accessed the online-format questionnaire via https://MDburnoutTH.med.cmu.ac.th (accessed on 30 April 2022). Data curation was conducted in a password-protected database, which was accessible only to the webmaster upon request by the principal investigator. Duplicate values were filtered and eliminated based on the birthdays of the participants. Posting study advertisements on websites, using the Line app for intern physicians’ instant messaging, and using social media sites like Facebook were all strategies employed to reach out to participants. Anonymous, voluntary, and informed consent was obtained from all participants in this study, with no identifying information collected. This study employed the concept of “consent by action”, which integrated informed consent into the participant’s interaction with the study website. Participants were introduced to the informed consent process upon their initial visit to the website’s first page, which served as the entry point to the questionnaire. The form provided detailed information about the study’s purpose, procedures, potential risks and benefits, confidentiality measures, and participants’ rights as research participants.

During the preparation of this work, we used ChatGPT 3.5 in order to check and correct grammatical errors during the manuscript writing process. After using this tool, we reviewed and edited the content as needed and take full responsibility for the content of the publication.

**Figure 1:**
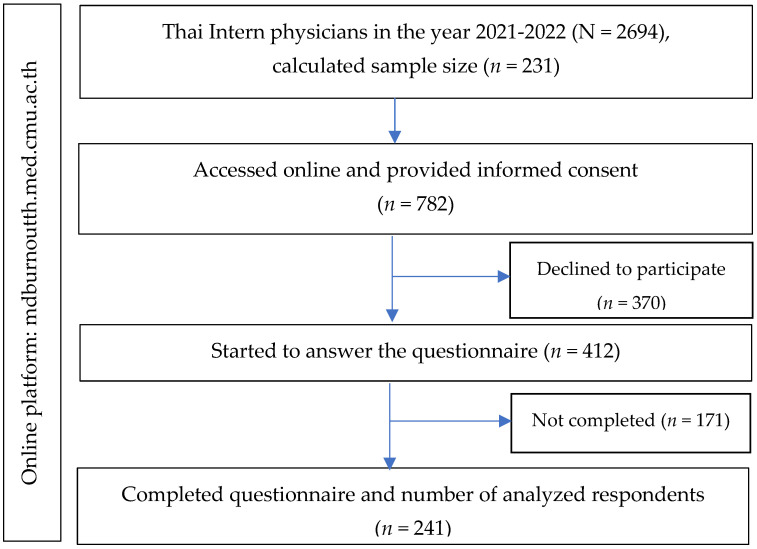
The study flow diagram of the recruitment of the participants.

The sample size was calculated using the n4Studies application to estimate the finite population proportion using the standard formula [47] based on the size of the population from which the sample was taken (the total number of Thai intern physicians working in the year 2021–2022 = 2694). Assuming a confidence level for the findings of 0.095, an expected proportion of 38.4% [33], an absolute precision of 5%, and a maximum error (d) of 0.06, we calculated the minimum sample size as 231. A total of 782 participants started filling in the questionnaire. Finally, 241 participants accessed the online platform to complete the survey. The flow diagram for participants is shown in Figure 1.

### 2.2. Measures

The data were collected from the self-administration online questionnaire, consisting of questions on the characteristics of study participants (7 items) (sex, age, monthly income, underlying diseases, medication, the region of hospital location, and hospital affiliation), burnout (22 items), and quality of work life (25 items).

#### 2.2.1. Burnout

Burnout was assessed using the Maslach Burnout Inventory—Human Services Survey for Medical Personnel, MBI-HSS (MP) [18]. This inventory consists of 22 items translated into Thai and has been used in previous studies [46], and the reliability of the scales was assessed using Cronbach’s alpha, with values as follows: EE subscale = 0.926; DP subscale = 0.811; and PA subscale = 0.833. Participants rated each item on a 7-point Likert scale, ranging from 0 to 6, based on the frequency of occurrence. The rating scale for the EE and PA subscales was as follows: “0 = Never, 1 = A few times a year or less, 2 = Once a month or less, 3 = A few times a month, 4 = Once a week, 5 = A few times a week, and 6 = Every day”. It is important to note that the PA subscale had an inverse rating score. In this study, Cronbach’s alpha values for the subscales were as follows: EE subscale = 0.946; DP subscale = 0.838; and PA subscale = 0.876 (Supplementary Table S1). The cut-points of the scores of EE, DP, and PA are presented in Table 1.

#### 2.2.2. Quality of Work Life

The QWL questionnaire used in this study was adapted from the Thai version of the Work-related Quality of Life Scale (TWRQLS) [25]. Initially, the WRQLS-2 comprised six factors and 34 items, which were subsequently refined to seven factors, including employee engagement (EET), resulting in a total of 32 items. The THWRQLS demonstrated reliability (Cronbach’s α of 0.671–0.82) and validity (Cronbach’s α of 0.92) in assessing the QWL among nurses in Thailand. However, it was noted to be time-consuming and had some content overlap [23]. Moreover, a seven-factor model derived from principal component factor analysis revealed that five items had factor loadings of less than 0.4, and content overlap was observed in some items [31]. Subsequently, Kongsin et al. developed the brief THWRQLS, retaining the seven dimensions but reducing the number of items to 25 from the original 32. Confirmatory factor analysis (CFA) indicated that most standardized factor loadings exceeded 0.5, with a χ^2^ goodness of fit of 268.772, a comparative fit index of 0.971, and a Cronbach’s alpha coefficient of 0.94. Most dimensions had coefficients greater than 0.7, with the exception of “SAW”, which scored 0.53 [25].

The THQWLS contains 25 questions in total. Each question uses a five-point Likert scale (1 = strongly disagree, 2 = disagree, 3 = neutral, 4 = agree, 5 = strongly agree), with four negative questions (reversed score): items 6, 8, 18, and 22. Of the total score, a good level was counted as a high WRQOL, and average or lower levels were counted as a low WRQOL. The cut-points of the scores are presented in Table 1. Cronbach’s coefficient alpha for the reliability of the overall QWL scale was 0.884. The reliability test of the seven QWL dimensions obtained a Cronbach’s coefficient alpha of between 0.62 and 0.82. The reliability tests for overall and QWL items in this study are shown in Supplementary Table S1, and the comparison of Cronbach’s coefficient alpha between this study and the validity study is shown in Supplementary Table S2.

### 2.3. Statistical Analysis

The statistical analysis was performed using STATA software version 16.0 (Stata Corp., College Station, TX, USA). A descriptive analysis determined the characteristics of the sample. The association between the burnout subscale and WRQOL with adjusted odds ratios (aOR) with 95% confidence intervals (95% CIs) was calculated by using multivariable binary logistic regression with backward stepwise selection. This study examined several variables, including burnout subscales, EE, DP, and PA—treated as determinants. The outcome variables comprised OVL and seven QWL dimensions, namely, EET, GWB, WCS, HWI, SAW, CAW, and JCS, which were analyzed using multivariable logistic regression. The assumptions necessary for logistic regression, such as the independence of errors, linearity in the logit for the continuous variables (e.g., age), the absence of multicollinearity, and the lack of strongly influential outliers, were verified. The influential outliers were handled with robust variance estimation. Directed acyclic graphs (DAGs) were utilized to clarify the set of confounders requiring adjustment and to identify factors associated with the outcome from the regression model. Specifically, DAGs assisted in determining the minimally adequate adjustment of confounding pathways, particularly concerning sex and age (see Supplementary Figure S1). All statistical tests were two-tailed, and a *p*-value of 0.05 was considered statistically significant. The findings of the study are reported according to the STROBE (Strengthening the Reporting of Observational Studies in Epidemiology) guidelines [48].

### 2.4. Ethical Considerations

This study was conducted following the Declaration of Helsinki guidelines, and the protocol was approved by the Research Ethics Committee of the Faculty of Medicine, Chiang Mai University, Thailand (Ethical number: 079/2022; date of approval: 24 February 2022).

## 3. Results

### 3.1. Descriptive Results on Quality-of-Work-Life Characteristics

This study distributed 412 questionnaires and received 241 completed responses. Table 2 outlines the participant characteristics and compares individuals reporting a moderate to high QWL and those reporting a low QWL. The gender distribution was relatively balanced, with 55.2% males and 44.8% females. The average age was 25.5 years (SD ± 2.01). Nearly half of the participants (46.9%) reported having underlying diseases, and among this group, 54.9% were taking medication. Most participants (83.0%) worked in hospitals under the Ministry of Public Health (83.0), while 17.0% worked in hospitals under other government sectors. Participants were located across different regions in Thailand, including central, northeast, north, south, and west regions, with varying proportions in each. Among the intern physicians surveyed, 72.6% reported a moderate to high QWL, while 27.4% reported a low QWL. Additionally, there are significant differences observed in the region of hospital location when comparing those with a moderate to high QWL and those with a low QWL (*p* = 0.015).

It is crucial to note that this study specifically targeted first-year intern physicians in Thailand. These individuals possess uniform characteristics and training levels, as they have all undergone the same six-year medical education program following high school graduation, which is supported by government-funded tuition. Upon the completion of their education, all graduates are obligated to work as physicians in provincial hospitals for a period of three years.

### 3.2. Quality of Work Life among Intern Physicians

**Figure 2:**
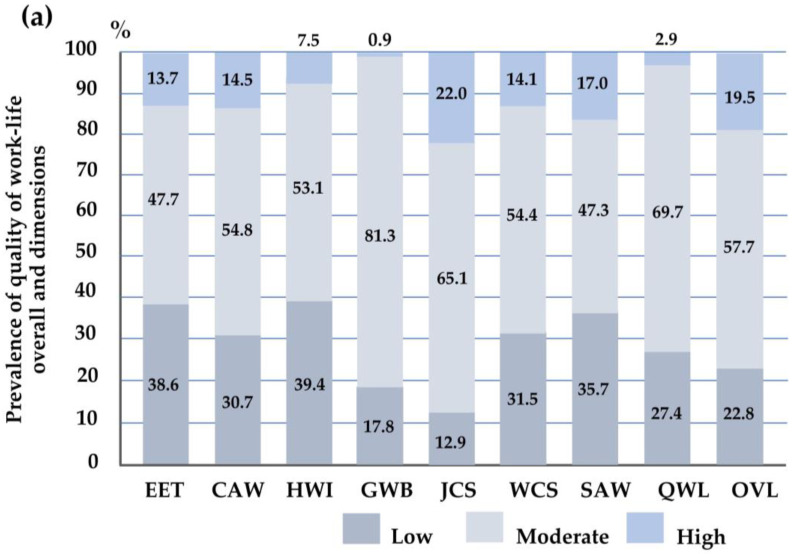

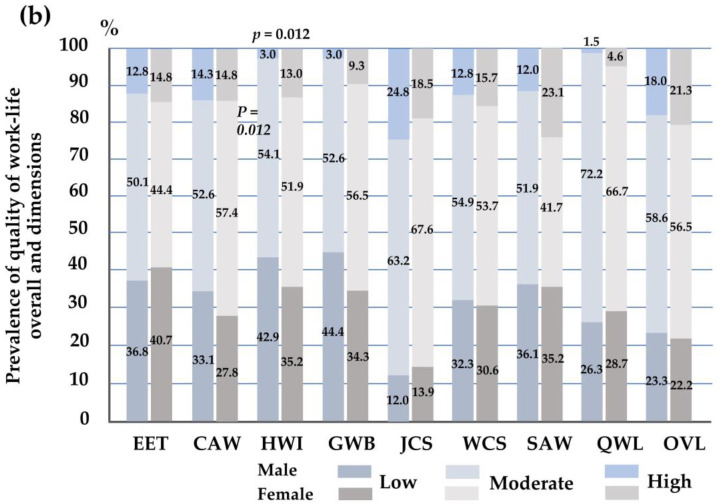
The histogram illustrates the proportions for (**a**) the quality of work life and its dimensions and (**b**) the quality of work life and all dimensions in males and females. The Chi-square test analyzed the differences between the two groups. Significant association at 0.05. Abbreviations: EET, employee engagement; CAW, control at work; HWI, home–work interface; GWB, general well-being; JCS, job and career satisfaction; WCS, working conditions; SAW, stress at work; QWL, quality of work life; OVL, overall quality of work life in response to a single question.

Figure 2 and Table S3 illustrate the QWL and its dimensions among intern physicians, with a comparison between males and females. Table S4 presents the frequency and proportion of QWL items. The majority of participants (69.7%) reported a moderate level of overall QWL, while 27.4% reported a low QWL, and only 2.9% reported a high QWL. Notably, 22.8% of participants reported a low level of overall quality of work life in response to a single question (OVL). Regarding specific QWL dimensions, the majority of participants indicated a low level of HWI (39.4%), followed by EET (38.6), SAW (35.7%), WCS (31.5%), and CAW (30.7%). Conversely, a considerable proportion reported low levels of JCS and GWB at 12.9% and 17.8%, respectively (Figure 2a). Females demonstrated a significantly higher proportion of QWL, particularly in the HWI dimension (*p* = 0.012). Interestingly, females experienced lower QWL levels in HWI compared to males (Figure 2b).

Additionally, the majority of both male and female participants reported moderate to high levels of overall QWL and OVL, with percentages of 73.7% and 76.6% for males and 71.3% and 77.8% for females, respectively. However, disparities emerge when examining specific QWL dimensions between the genders. Among males, a significant proportion reported low levels of GWB (44.4%), HWI (42.9%), EET (36.8%), SAW (36.1%), CAW (33.1%), and WCS (32.3%). Conversely, a smaller percentage reported low levels of JCS (12.0%). For females, the majority reported low levels of EET (40.7%), followed by HWI and GWB (both at 35.2%), WCS (30.6%), and CAW (27.8%). The proportion of females reporting low JCS was 13.9%. Notably, females demonstrated a significantly lower proportion of HWI dimension (*p* = 0.012) compared to males. This indicates females experienced lower levels of HWI compared to males, as illustrated in Figure 2b.

### 3.3. Burnout Subscales among Intern Physicians

**Figure 3:**
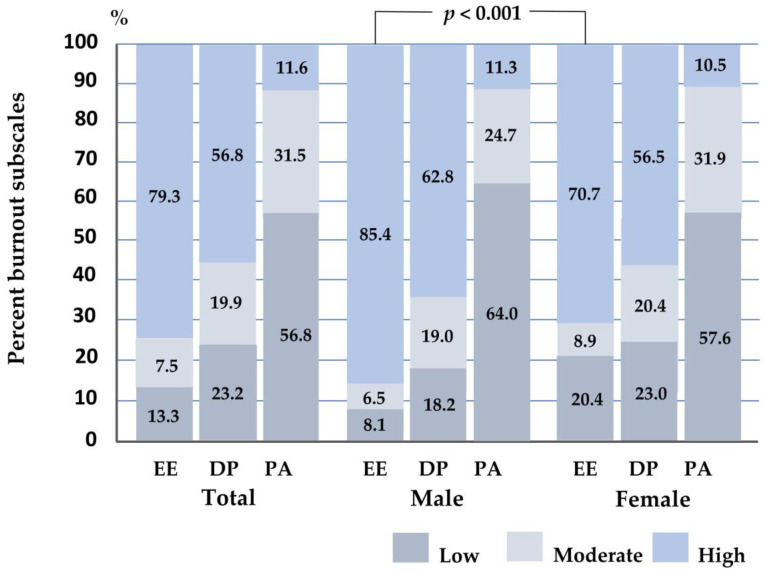
The histogram illustrates the proportion of burnout subscale scores among participants and classified by gender. The Chi-square test analyzed the differences between the two groups. Significant association at 0.001. EE, emotional exhaustion is classified as low ≤ 16 points, moderate = 17–26 points, and high > 26 points; DP, depersonalization is classified as low ≤ 6 points, moderate = 7–12 points, and high > 12 points; PA, personal accomplishment is classified as low ≤ 31 points, moderate = 32–38 points, and high > 38 points.

Figure 3 and Table S3 offer insights into the proportion of burnout subscale scores among intern physicians, including a comparison between males and females. The findings indicate that a significant majority of participants (79.3%) experienced a high level of EE, while 13.3% reported a low level of EE. Moreover, 56.8% of them experienced both high levels of DP and low PA. Additionally, 19.9% reported moderate levels of DP, and 31.5% reported moderate levels of PA. Furthermore, 23.2% indicated low levels of DP, while 11.6% reported high levels of PA. Notably, males experienced a significantly higher proportion of high levels of EE (85.4%) compared to females (70.7%) (*p* < 0.001). Males also reported higher levels of DP (62.8%) compared to females (56.5%). Additionally, 64.0% of males reported low levels of PA, which was a higher proportion than females (57.6%).

Regarding the distribution of burnout subscales by sex, the majority of both males and females experienced high levels of EE, with proportions of 85.4% and 70.7%, respectively. Notably, a higher percentage of females reported low levels of EE compared to males, with proportions of 20.4% and 8.1%, respectively. This discrepancy in EE levels in males and females was statistically significant (*p* < 0.001). Additionally, males exhibited higher levels of DP compared to females, with proportions of 62.8% and 56.5%, respectively. Conversely, the percentage of females reporting low levels of DP exceeded that of males, with proportions of 23.0% and 18.2%, respectively. Furthermore, 64.0% of males reported low levels of PA, a higher proportion than females (57.6%). However, males also reported a higher proportion of high PA compared to females, with percentages of 11.3% and 10.5%, respectively (see Figure 3).

### 3.4. Associations between Burnout and Quality of Work Life among Intern Physicians

Table 3, Table 4 and Table 5 present the results of exploratory models utilizing multivariable logistic regression. These models aimed to determine the associations between burnout subscales, overall QWL, and individual QWL dimensions. The analyses were adjusted for potential confounders such as gender and age. The analyses were adjusted for potential confounders, including sex and age. However, it is important to acknowledge that the study’s results may still be influenced by residual confounding factors. For instance, unmeasured work-related stressors could affect burnout and QWL. Additionally, acute or dynamic events occurring during the study period, such as sudden increases in workload, post-shift fatigue, workplace accidents, or seasonal variations in workload and environmental factors, might lead to short-term fluctuations in burnout and QWL measures. These factors could complicate the interpretation of the findings.

We analyzed the associations using three models: Model 1 focused on the association between burnout subscales, overall QWL, and OVL. Model 2 investigated the association of burnout subscales with QWL dimensions, including EET, GWB, and WCS. Model 3 explored the association between burnout subscales and QWL dimensions, including HWI, SAW, CAW, and JCS.

#### 3.4.1. Model 1: Associations between Burnout Subscales and Overall QWL

Model 1 demonstrated that intern physicians experiencing high levels of DP were more likely to have a low QWL (aOR 2.08, 95% CI 1.01 to 4.31, *p* = 0.048) compared to those with low to moderate levels of DP. However, DP levels were not associated with OVL. Moreover, participants with low levels of PA were significantly associated with both a low QWL (aOR 2.74, 95% CI 1.04 to 5.39, *p* = 0.003) and a low OVL (aOR 2.95, 95% CI 1.40 to 6.23, *p* = 0.005) compared to those with moderate to high levels of PA. Notably, EE levels were not associated with either QWL or OVL (Table 3).

**Table 3:** The association between burnout subscales and the overall quality of work life.

QWL (*n* = 227)	High Emotional Exhaustion (EE)				High Depersonalization (DP)				Low Personal Accomplishment (PA)			
	aOR	SE	95% CI	*p*	aOR	SE	95% CI	*p*	aOR	SE	95% CI	*p*
Low QWL ^a^	1.04	0.52	0.38 to 2.79	0.945	2.08	0.77	1.01 to 4.31	0.048 *	2.74	0.94	1.40 to 5.39	0.003 *
Low OVL ^a^	1.17	0.63	0.41 to 3.34	0.763	1.31	0.50	0.62 to 2.76	0.483	2.95	1.12	1.40 to 6.23	0.005 *

^a^ Exploratory model using multivariable logistic regression analysis adjusted for sex and age. Reference groups are moderate to high levels of QWL and OVL, low to moderate levels of EE and DP, and moderate to high PA levels. * Significant association at 0.05. Abbreviations: QWL, quality of work life; OVL, overall quality of work life in response to a single question; aOR, adjusted odds ratio; SE, standard error; CI, confidence interval.

#### 3.4.2. Model 2: Associations between Burnout Subscales and QWL Dimensions, Including EET, GWB, and WCS

Table 4 examines participants’ perceptions of intrinsic factors related to QWL, specifically EET, GWB, and WCS. Model 2 shows that intern physicians with low levels of PA were more likely to report low EET (aOR 3.24, 95% CI 1.78 to 5.88, *p* < 0.001) compared to those with moderate to high PA levels. However, DP and EE levels were not found to be associated with EET. Additionally, participants with high EE levels (aOR 2.82, 95% CI 1.04 to 7.64, *p* = 0.042) and low PA levels (aOR 2.30, 95% CI 1.28 to 4.12, *p* = 0.005) were significantly associated with low GWB compared to those with low to moderate levels of EE and moderate to high PA. DP levels were not significantly associated with GWB. Furthermore, participants with high DP levels (aOR 2.03, 95% CI 1.04 to 3.99, *p* = 0.039) and low PA levels (aOR 2.32, 95% CI 1.25 to 4.31, *p* = 0.008) were significantly associated with low WCS compared to those with low to moderate levels of DP and moderate to high PA. Notably, EE levels did not show significance with WCS.

**Table 4:** The association between burnout subscales and QWL dimensions, including EET, GWB, and WCS.

QWL Dimensions (*n* = 227)	High Emotional Exhaustion (EE)				High Depersonalization (DP)				Low Personal Accomplishment (PA)			
	aOR	SE	95% CI	*p*	aOR	SE	95% CI	*p*	aOR	SE	95% CI	*p*
Low EET ^a^	1.71	0.78	0.70 to 4.17	0.238	1.37	0.44	0.73 to 2.56	0.327	3.24	0.99	1.78 to 5.88	<0.001 **
Low GWB ^a^	2.82	1.43	1.04 to 7.64	0.042 *	1.81	0.58	0.97 to 3.38	0.062	2.30	0.68	1.28 to 4.12	0.005 *
Low WCS ^a^	1.36	0.67	0.52 to 3.58	0.534	2.03	0.70	1.04 to 3.99	0.039 *	2.32	0.73	1.25 to 4.31	0.008 *

^a^ Exploratory model using multivariable logistic regression analysis adjusted for sex and age. Reference groups are moderate to high levels of EET, GWB, and WCS as QWL dimensions, low to moderate levels of EE and DP, and moderate to high PA levels. * Significant association at 0.05, ** Significant association at 0.001. Abbreviations: QWL, quality of work life; EET, employee engagement; GWB, general well-being; WCS, working conditions; aOR, adjusted odds ratio; SE, standard error; CI, confidence interval.

#### 3.4.3. Model 3: Associations between Burnout Subscales and QWL Dimensions Including HWI, SAW, CAW, and JCS

Table 5 explores participants’ perceptions of extrinsic factors and working conditions related to QWL, specifically HWI, SAW, CAW, and JCS. Model 3 reveals that intern physicians with high EE levels (aOR 8.56, 95% CI 2.19 to 33.51, *p* = 0.002) and high DP levels (aOR 2.21, 95% CI 1.12 to 4.02, *p* = 0.021) were significantly associated with low SAW compared to those with low to moderate levels of EE and DP. However, PA did not show an association with SAW. Furthermore, participants with low PA levels (aOR 3.64, 95% CI 1.88 to 7.05, *p* = < 0.001) were significantly associated with low CAW compared to those with moderate to high PA levels. Interestingly, EE, DP, and PA levels did not exhibit significant associations with HWI and JCS.

**Table 5:** The association between burnout subscales and QWL dimensions, including HWI, SAW, CAW, and JCS.

QWL Dimensions (*n* = 227)	High Emotional Exhaustion (EE)				High Depersonalization (DP)				Low Personal Accomplishment (PA)			
	aOR	SE	*p*	95% CI	aOR	SE	*p*	95% CI	aOR	SE	*p*	95% CI
Low HWI ^a^	2.32	1.06	0.067	0.94 to 5.69	1.03	0.31	0.930	0.56 to 1.87	1.01	0.29	0.974	0.58 to 1.76
Low SAW ^a^	8.56	5.96	0.002 *	2.19 to 33.51	2.12	0.69	0.021 *	1.12 to 4.02	0.90	0.27	0.721	0.50 to 1.61
Low CAW ^a^	1.35	0.65	0.537	0.52 to 3.46	1.26	0.43	0.499	0.65 to 2.45	3.64	1.23	<0.001 **	1.88 to 7.05
Low JCS ^a^	0.31	0.19	0.059	0.09 to 1.04	1.97	1.12	0.232	0.65 to 5.97	2.72	1.49	0.066	0.94 to 7.93

^a^ Exploratory model by multivariable logistic regression analysis adjusted for sex and age. Reference groups are moderate to high levels of HWI, SAW, CAW, and JCS as QWL dimensions, low to moderate levels of EE and DP, and moderate to high PA levels. * Significant association at 0.05, ** significant association at 0.001. Abbreviations: QWL, quality of work life; HWI, home–work interface; SAW, stress at work; CAW, control at work; JCS, job and career satisfaction; aOR, adjusted odds ratio; SE, standard error; CI, confidence interval.

## 4. Discussion

This study is the first investigation into the QWL and the association between burnout and QWL among intern physicians in Thailand. Considering the unique demands of medical training and the transition into early-career practice, comprehending the factors influencing QWL, particularly burnout, holds importance. Such insights are essential for developing tailored interventions and policies aimed at enhancing QWL and mitigating burnout among intern physicians. Our findings show that a significant proportion of intern physicians assessed their overall QWL as moderate or low, with 69.7% and 27.4%, respectively. This differs from previous studies conducted in Thailand using the same structure assessment tool, where medical residents [31] and university physicians [32] reported lower rates of low QWL. Specifically, Thai physicians in public hospitals reported a poor QWL at a rate of 67.5% [33]. Most interns rated HWI and EET as low, while SAW received low ratings from most residents and university physicians. However, interns rated all QWL dimensions higher than their counterparts, with HWI and EET having a higher proportion of low ratings (Table 6). This study found that intern physicians’ low ratings were largely due to the COVID-19 pandemic, which likely increased stress and workload, leading to lower QWL scores compared to other studies. Moreover, our study focused on interns actively practicing in tertiary public hospitals throughout the first and second waves of the COVID-19 pandemic in Thailand, where infection rates were notably high [49]. Previous studies in China and Poland have also reported similar trends, with young doctors experiencing low QWL levels [21,50]. It is important to acknowledge that there is variation in the prevalence of QWL, which may underscore the importance of addressing QWL issues among early-career physicians and emphasize the necessity of sharing best practices and strategies to support the QWL of healthcare professionals worldwide.

Our findings reveal that burnout, specifically EE, DP, and reduced PA, significantly impacts the QWL experienced by physicians during their internship. This effect is evident across all dimensions of QWL except for HWI and JSC. There is no significant relationship between EE and overall QWL. These findings emphasize the importance of targeting specific aspects of burnout to enhance physicians’ work-life quality effectively. Previous international studies have consistently demonstrated an association between burnout levels among physicians and various aspects of QWL, including career engagement, job satisfaction, working conditions, work–life balance or integration, and overall well-being [15,41,42,43,51,52,53]. Additionally, burnout has been associated with detrimental outcomes such as low professionalism, turnover intention, early retirement, and career change among physicians [42,51,54]. These findings suggest the importance of addressing burnout and QWL issues within the healthcare sector. Despite variations in social contexts and organizational cultures, the defining characteristics of burnout and its profound impact on both professional and personal domains remain evident. Notably, an individual’s sense of efficacy and fulfillment significantly influences their effectiveness and well-being as physicians. Moreover, the consequences of burnout extend beyond the individual level, affecting patient care. Factors such as decreased effectiveness, loss of professional precision, and diminished compassion can compromise the quality and safety of clinical care, ultimately leading to adverse outcomes for patients and contributing to a low QWL in physicians [54,55,56,57,58]. Unsurprisingly, frequent encounters with burnout are a significant predictor of a low QWL in all domains, except the HWI and JSC dimensions.

This study found that high rates of low PA, a subscale of burnout, were associated with low levels of EET among intern physicians, aligning with findings from a previous study [58]. This suggests that low EET may contribute to decreased well-being and performance among these professionals [59]. PA is an individual’s subjective evaluation of their competence, productivity, and effectiveness in their work [18], while EET is the level of enthusiasm for their work, their passion, and their motivation to contribute to the organization’s success. Engagement can be viewed as the opposite of burnout, characterized by dedication, vigor, and absorption in one’s work [60]. High PA levels indicate a strong sense of competence, achievement, and satisfaction, while low levels suggest feelings of inadequacy and diminished satisfaction [61]. To enhance intern engagement during their early-career practice, training programs should prioritize professional development initiatives. This may include offering continuing education courses aimed at developing leadership skills and knowledge. By empowering interns to make decisions and encouraging autonomy and responsibility in their work, training programs can potentially increase their sense of PA.

This study found that burnout among intern physicians significantly impacts their general well-being (GWB), with high EE and low PA being key factors contributing to lower levels of GWB. These factors, including life satisfaction, happiness, strong social support, and fulfillment [62], are crucial for aspects such as patient safety and work engagement [41]. EE, or feelings of emotional depletion from work-related stressors, and reduced PA, reflecting reduced efficacy and decreased professional satisfaction, significantly influence interns’ QWL assessments [18]. Healthcare organizations have the potential to decrease EE and increase PA among interns, ultimately fostering higher levels of GWB. This can be achieved by actively promoting self-care and leisure activities among interns, as well as providing resources such as mindfulness meditation, relaxation techniques, and stress reduction exercises. Additionally, recognizing interns’ achievements through programs, awards, and incentives can further contribute to their overall well-being [63].

This study found a significant association between high DP, low PA, and lower levels of WCS, particularly in high-stress environments such as hospitals. Working conditions refer to the organization, including environments and psycho-social conditions that affect QWL. DP involves developing negative attitudes toward patients, colleagues, and the healthcare system and can increase stress among intern physicians [18]. Poor working conditions, such as excessive workload, long hours, lack of autonomy, inadequate support from supervisors or colleagues, and organizational factors such as insufficient resources or ineffective policies, were associated with burnout by increasing stress levels and undermining employees’ sense of fulfillment and engagement [64,65]. Improving intern working conditions necessitates addressing high levels of DP and low levels of PA. This can be accomplished through collaborative efforts among leaders, mentors, and healthcare professionals. It is essential to ensure adequate staffing, resources, and support for interns, along with providing regular supervision and feedback from experienced mentors.

High levels of EE and DP, as burnout dimensions, significantly affect the SAW of intern physicians, influencing their ability to effectively manage stress in the demanding healthcare environment. EE, characterized by feelings of emotional depletion and exhaustion, can lead to increased stress levels as they navigate challenging patient cases, long work hours, and high-performance pressures [18]. DP, such as negative interpersonal interactions and a sense of detachment from work, can create additional strain and tension in the workplace, exacerbating feelings of stress and burnout [18]. To mitigate EE and DP among interns and reduce workplace stress, healthcare organizations can implement several strategies. These include adjusting the workload distribution to prevent excessive work hours and overload, offering stress management workshops to equip interns with coping mechanisms, and cultivating a supportive environment. By doing so, interns are encouraged to connect with their peers, share experiences, and seek assistance and support as needed [61,66].

Our findings revealed that 30.7% of interns had low levels of CAW, possibly due to the higher authority of hospital staff or residents in decision-making processes within the hospital environment. A positive correlation was observed between PA and CAW among intern physicians, highlighting the importance of autonomy and decision-making authority in influencing interns’ competence and achievement. A lack of control and autonomy in the workplace has been identified as a significant factor contributing to burnout among intern physicians [67,68]. CAW, which refers to an individual’s perceived ability to make decisions and influence their work environment, can empower individuals to overcome challenges and achieve goals, contributing to higher job satisfaction and overall well-being among the workforce [61]. To increase PA among interns, targeted interventions should focus on enhancing competence, achievement, and fulfillment. This can be achieved through hands-on experience and clinical rotations, providing interns with opportunities to apply their skills in real-world settings and develop mastery and competence in their abilities.

Our findings found no association between burnout, HWI, and JCS among intern physicians. This could be due to differences in the context and stressors experienced in academic versus professional settings. Thai intern physicians, as early medical graduates, face high-pressure environments where they are responsible for frontline patient care, including handling the emergency department, which requires training and creates emotional challenges. In academic settings, the stressors may be more related to patient care responsibilities, workplace dynamics, and clinical pressure. These will contribute to burnout [66]. On the other hand, the home–work interface stress in academic settings may be more focused on academic performance, deadlines, and balancing academic responsibilities with other life aspects [22]. In certain healthcare settings, burnout may be perceived as a normal part of training and professional development. Healthcare organizations should prioritize identifying and addressing additional factors that impact interns’ job satisfaction and their ability to maintain a healthy work–life balance. The regular monitoring of trends can facilitate ongoing improvement efforts in enhancing overall job satisfaction and work–life balance among interns.

This study provides valuable insights into the association between burnout and QWL among intern physicians in Thailand, but several limitations should be considered when interpreting the findings. Firstly, the cross-sectional design of the study limits the ability to establish causality relationships between burnout and QWL dimensions. Although we can identify associations between these variables, we cannot determine the direction of causality or rule out the possibility of reverse causation. Future longitudinal studies are needed to explore the dynamic interplay between burnout and QWL over time, allowing for a more nuanced understanding of their relationship. Secondly, the use of self-report measures to assess burnout and QWL may introduce social desirability bias or response bias. Participants might have underreported or overreported their experiences, leading to potential measurement errors. Integrating objective measures or combining self-report assessments with other methods, such as observer ratings or qualitative interviews, could provide a more comprehensive understanding of burnout and QWL among intern physicians.

Thirdly, the convenience sampling method employed in this study may have introduced selection bias, as participants who volunteered to take part may differ systematically from those who did not. This could affect the generalizability of the findings to the broader population of intern physicians in Thailand. Future research should aim to utilize more representative sampling methods, such as random or stratified sampling, to enhance the generalizability of results. Lastly, this study might not have accounted for all potential confounding factors influencing QWL among intern physicians. Variables such as significant life events, specific medical conditions, or individual coping mechanisms could substantially impact QWL but were not explicitly measured in this study. Future research should aim to comprehensively identify and assess these additional factors to provide a more nuanced understanding of their influence on QWL outcomes among healthcare professionals.

It is essential to acknowledge the potential limitations in generalizing the findings of other cultural contexts. Socio-cultural factors, organizational structures, and healthcare systems vary significantly across countries, profoundly impacting the experiences of intern physicians regarding burnout and QWL. Thailand’s unique cultural context, characterized by collectivism, respect for authority, and a strong emphasis on interpersonal relationships [69], can influence how burnout and QWL are reported and managed among intern physicians. The hierarchical structure within healthcare organizations and the emphasis on maintaining harmonious relationships may also affect how intern physicians cope with work-related stressors and navigate interpersonal dynamics. Therefore, cross-cultural research is imperative to enhance the generalizability of findings and provide a more comprehensive understanding of these phenomena among intern physicians globally.

## 5. Conclusions

In Thailand, intern physicians working in public hospitals frequently experienced a significantly higher prevalence of low QWL, particularly in dimensions such as HWI and EET. Similar trends were observed during the COVID-19 pandemic in China and Poland [21,50]. This low QWL is strongly associated with burnout subscales, particularly high DP and low PA among intern physicians. This association is evident across all dimensions of QWL except for HWI and JSC. To address these challenges, healthcare organizations should conduct regular surveys of intern physicians to assess their QWL and burnout levels. These data can inform targeted interventions and policy recommendations. Hospitals should prioritize various dimensions of QWL, including EET, GWB, WCS, SAW, CAW, and burnout subscales. Ensuring reasonable work hours and adequate resources is crucial for preventing burnout and improving QWL, which may require scheduling adjustments, sufficient staffing, and the fostering of open communication. Healthcare organizations should create support systems tailored to the unique needs of intern physicians., including employee assistance programs, leadership training, professional development opportunities, and wellness initiatives. Employing cost–benefit analysis can guide resource allocation in Thai hospitals, ensuring the effectiveness of interventions. Moreover, hospitals must address scheduling flexibility challenges and find creative solutions to accommodate interns’ schedules to ensure successful intervention implementation.

Further research is needed to comprehend the dynamic relationship between burnout and QWL among intern physicians, including longitudinal studies to explore factors contributing to these changes over time. Additionally, examining burnout and QWL by sex can inform targeted interventions, support systems, and professional development initiatives aimed at sustaining a resilient healthcare workforce. Cross-cultural research is also imperative for developing culturally sensitive interventions.

## Figures and Tables

**Table 1:** The cut-points of the scores of quality of work life and its subscales and burnout subscales.

Quality of Work Life (QWL) Subscales [Number of Items]	Levels of QWL		
	Low (Points)	Moderate (Points)	High (Points)
Employee engagement (EET) [2]	2 to 4	5 to 7	8 to 10
Control at work (CAW) [3]	3 to 6	7 to 10	11 to 15
Home–work interface (HWI) [3]	3 to 6	7 to 10	11 to 15
General well-being (GWB) [4]	4 to 9	10 to 15	16 to 20
Job and career satisfaction (JCS) [6]	6 to 13	14 to 21	22 to 30
Working conditions (WCS) [3]	3 to 6	7 to 10	11 to 15
Stress at work (SAW) [3]	3 to 6	7 to 10	11 to 15
Total scores of QWL [24]	24 to 56	57 to 89	90 to 120
Overall (OVL) [1]	1	2 to 3	4 to 5
**Burnout Subscales** **[Number of Items]**	**Levels of Burnout**		
	**Low (Points)**	**Moderate (Points)**	**High (Points)**
Emotional exhaustion (EE) [9]	0 to 16	17 to 26	>26
Depersonalization (DP) [5]	0 to 6	7 to 12	>12
Personal accomplishment (PA) [8]	>38	32 to 38	0 to 31

**Table 2:** Characteristics of intern physicians according to quality of work life.

Characteristics	Total (*n* = 241)	Quality-of-Work-Life Levels		*p*-Value
		Moderate to High (*n* = 175, 72.6%)	Low (*n* = 66, 27.4%)	
Sex				
Female	108 (44.8)	77 (44.0)	31 (47.0)	0.772 ^a^
Male	133 (55.2)	98 (56.0)	35 (53.0)	
Age (years)	25.52 (2.01)	25.54 (1.93)	25.55 (2.18)	0.960 ^b^
Income per month (Baht), mean ± SD	60,937.89 ±17,054.66	62,482.56 ±17,762.72	60,000 ±13,017.08	0.340 ^b^
Underlying diseases				
Yes	113 (46.9)	82 (46.9)	31 (47.0)	0.988 ^a^
No	128 (53.1)	93 (53.1)	35 (53.0)	
Taking medication (n = 113)				
Yes	62 (54.9)	47 (75.8)	15 (24.2)	0.513 ^a^
No	51 (45.1)	35 (68.6)	16 (31.4)	
Region of hospital location				
Central	68 (28.2)	44 (25.1)	24 (36.4)	0.015 ^a,^*
East	24 (10.0)	23 (13.1)	1 (1.5)	
West	17 (7.1)	10 (5.7)	7 (10.6)	
North	34 (14.1)	29 (16.6)	5 (7.6)	
Northeast	65 (27.0)	44 (25.1)	21 (31.8)	
South	33 (13.7)	25 (14.3)	8 (12.1)	
Hospital affiliation				
Ministry of Public Health	200 (83.0)	145 (82.9)	55 (83.3)	0.930 ^b^
Others	41 (17.0)	30 (17.1)	11 (16.7)	

Statistical analysis with ^a^ Chi-square test, ^b^ Fisher’s Exact test. * Significant association at 0.05. Abbreviations: Baht, Thai Baht; SD, standard deviation.

**Table 6:** Comparison to previous studies in Thai physicians for quality of work life and its dimension levels.

	Quality of Work Life and Its Dimensions in Thai Physicians, %								
	Low Levels			Moderate Levels			High Levels		
	Residents (2015) ^a^	University Physicians (2019) ^b^	Interns (2022) ^c^	Residents (2015) ^a^	University Physicians (2019) ^b^	Interns (2022) ^c^	Residents (2015) ^a^	University Physicians (2019) ^b^	Interns (2022) ^c^
QWL ^≠^	1.5	3.0	27.4	76.6	58.6	69.7	21.9	38.4	2.9
EET	0.8	3.5	38.6	55.5	42.1	47.7	43.8	54.5	13.7
CAW	15.9	3.5	30.7	56.2	51.8	54.8	27.3	44.7	14.5
HWI	23.4	6.1	39.4	52.7	54.5	53.1	23.4	39.5	7.5
GWB	5.9	5.6	17.8	73.4	46.4	81.3	20.7	47.9	0.9
JSC	0.0	0.9	12.9	36.7	22.1	65.1	63.3	77.0	22.0
WCS	9.8	3.0	31.5	69.5	54.2	54.4	20.7	42.7	14.1
SAW	35.9	20.2	35.7	54.7	67.7	47.3	9.4	12.2	17.0

^≠^ QWL, total scores of quality of work life; ^a^ a study in Thai residents in 2015 (*n* = 256) [31]; ^b^ a study in Thai university doctors in 2017 (*n* = 339) [32]; ^c^ this study among intern physicians in 2022 (*n* = 241). Abbreviations: EET, employee engagement; CAW, control at work; HWI, home–work interface; GWB, general well-being; JCS, job and career satisfaction; WCS, working conditions; SAW, stress at work.

## Data Availability

The data presented in this study are available from the corresponding author on reasonable request.

## Supplementary Materials

The following are available online at https://www.mdpi.com/article/10.3390/bs14050361/s1: Table S1: Quality-of-work-life questionnaire reliability assessment; Table S2: The reliability test of seven QWL subscales; Table S3: Quality-of-work-life scales and burnout subscales among intern physicians; Table S4: Frequency and percentage of quality-of-work-life levels among intern physicians. Figure S1: The directed acyclic graph (DAG) concerning the statistical modeling of multivariable logistic regression was generated with publication licensed by BioRender, Toronto, Canada (Agreement number: QG237SORHP, 19 November 2021).
