# Supplementary material for: Burnout and Quality of Work Life among Physicians during Internships in Public Hospitals in Thailand
Source: Behav Sci (Basel). 2024 Apr 25;14(5):361. doi: 10.3390/bs14050361 (PMC11117651; doi:10.3390/bs14050361)
Supplement: Supplementary file 1 [file behavsci-14-00361-s001.zip › Supplementary Figure S1.pdf]

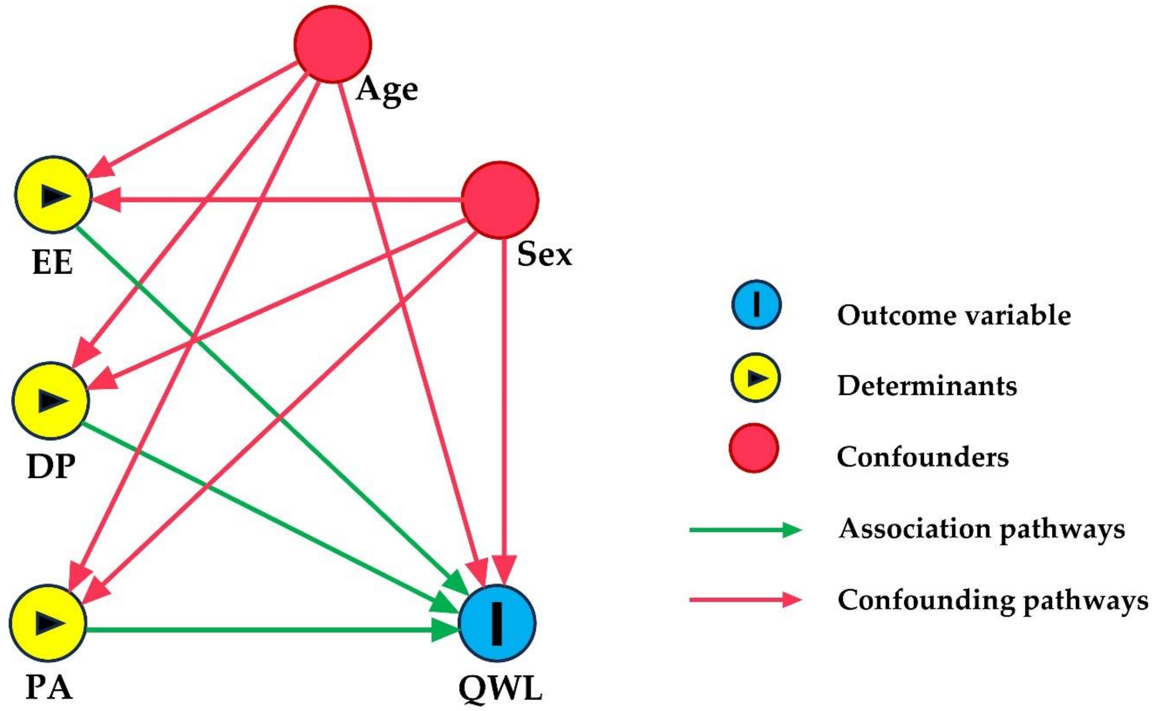

Supplementary Figure S1. The directed acyclic graph (DAG), concerning the statistic modeling of multivariable logistic regression, was generated with publication licensed by BioRender, Toronto, Canada (Agreement number: QG237SORHP, 19 November 2021). Green arrows indicate the significant association pathways; red arrows represent confounding pathways; red ellipses represent confounders; blue ellipses represent the dependent variable; yellow ellipses represent determinants; and blue ellipses represent the outcome variable.
