# Supplementary material for: Burnout and Quality of Work Life among Physicians during Internships in Public Hospitals in Thailand
Source: Behav Sci (Basel). 2024 Apr 25;14(5):361. doi: 10.3390/bs14050361 (PMC11117651; doi:10.3390/bs14050361)
Supplement: Supplementary file 1 [file behavsci-14-00361-s001.zip › Supplementary Table S1.pdf]

**Table S1.** The reliability test of seven quality of work-life (QWL) subscales.

| Subscales | Items | Cronbach's Alpha | |
|---|---|---|---|
| | | Validation study* | Present study |
| Total scores of QWL | 24 | 0.94 | 0.88 |
| (1) Employee engagement (EET) | 2 | 0.80 | 0.82 |
| (2) Control at work (CAW) | 3 | 0.73 | 0.76 |
| (3) Homework interface (HWI) | 3 | 0.74 | 0.69 |
| (4) General well-being (GWB) | 4 | 0.80 | 0.62 |
| (5) Job and career satisfaction (JCS) | 6 | 0.78 | 0.74 |
| (6) Working conditions (WCS) | 3 | 0.71 | 0.71 |
| (7) Stress at work (SAW) | 3 | 0.53 | 0.62 |

*The brief Thai version of the work-related quality of life scale (Brief THWRQLS) [39]
