# Supplementary material for: Burnout and Quality of Work Life among Physicians during Internships in Public Hospitals in Thailand
Source: Behav Sci (Basel). 2024 Apr 25;14(5):361. doi: 10.3390/bs14050361 (PMC11117651; doi:10.3390/bs14050361)
Supplement: Supplementary file 1 [file behavsci-14-00361-s001.zip › Supplementary Table S2.pdf]

Table S2. Quality of work-life questionnaire reliability assessment.

Questions	Sign	Mean	SD	Corrected Item-Total Correlation	Cronbach's Alpha if Item Deleted
1. I have a clear set of goals and purposes in doing my job.	+	3.30	1.16	-0.265	0.900
2. I feel able to voice opinions and influence changes in my area of work.	+	2.68	1.18	0.537	0.877
3. I have the opportunity to use my abilities at work.	+	3.38	1.04	0.543	0.877
4. My employer provides adequate facilities and flexibility for me to fit work around my family life.	+	2.59	1.07	0.598	0.876
5. My current working hours/ patterns suit my personal circumstances.	+	2.14	0.96	0.587	0.876
6. I often feel under pressure at work.	-	2.34	1.17	-0.020	0.892
7. When I have done a good job, it is acknowledged by my line manager.	+	2.96	1.19	0.557	0.876
8. Recently, I have been feeling unhappy and depressed.	-	2.71	1.26	0.120	0.889
9. I am encouraged to develop new skills.	+	2.93	1.07	0.619	0.875
10. I am involved in decisions that affect me in my own area of work.	+	2.81	1.14	0.597	0.875
11. My employer provides me with what I need to do my job effectively.	+	2.38	1.04	0.635	0.875
12. In most ways, my life is close to ideal.	+	1.98	0.92	0.628	0.876
13. I work in a safe environment.	+	2.79	1.11	0.630	0.875
14. Generally, things work out well for me.	+	2.80	0.98	0.754	0.872
15. I am satisfied with the career opportunities available for me here.	+	2.85	1.13	0.716	0.872
16. I am satisfied with the training I received in order to perform my present job.	+	2.99	1.15	0.683	0.873
17. Recently, I have been feeling reasonably happy all things considered.	+	2.80	1.07	0.622	0.875
18. I have an unachievable deadline.	-	2.77	1.23	-0.077	0.874
19. I am able to achieve a healthy balance between my work and home life.	+	2.46	1.08	0.589	0.876
20. I am proud to tell others that I am part of this organization.	+	2.70	1.11	0.650	0.874
21. I would recommend this organization as a good one to work for.	+	2.39	1.19	0.663	0.873
22. I am pressured to work long hours.	-	2.69	1.35	-0.161	0.898
23. I have sufficient opportunities to question managers about the changes at work.	+	2.22	1.10	0.702	0.873
24. I am happy with the physical environment where I usually work.	+	2.62	1.10	0.700	0.870
Test scale (Cronbach's alpha coefficient 24 items)					0.884
