# Supplementary material for: Burnout and Quality of Work Life among Physicians during Internships in Public Hospitals in Thailand
Source: Behav Sci (Basel). 2024 Apr 25;14(5):361. doi: 10.3390/bs14050361 (PMC11117651; doi:10.3390/bs14050361)
Supplement: Supplementary file 1 [file behavsci-14-00361-s001.zip › Supplementary Table S3.pdf]

Table S3. Quality of work-life (QWL) scales and burnout subscales among intern physicians.

QWL subscales (*n* = 241)	Min-Max	Mean ±SD	Median [IQR]	QWL levels, n (%)		
				Low	Moderate	High
Employee engagement (EET)	2 - 10	5.09 ±2.12	5.0 [4.0]	93 (38.6)	115 (47.7)	33 (13.7)
Control at work (CAW)	3 - 15	7.71 ±2.80	8.0 [4.0]	74 (30.7)	132 (54.8)	35 (14.5)
Homework interface (HWI)	3 - 15	7.18 ±2.44	7.0 [3.0]	95 (39.4)	128 (53.1)	18 (7.5)
General well-being (GWB)	4 - 20	10.29 ±2.91	10.0 [4.0]	40 (17.8)	183 (81.3)	2 (0.9)
Job and career satisfaction (JCS)	6 - 29	18.42 ±4.44	19.0 [5.0]	31 (12.9)	157 (65.1)	53 (22.0)
Working conditions (WCS)	3 - 15	7.79 ±2.57	8.0 [4.0]	76 (31.5)	131 (54.4)	34 (14.1)
Stress at work (SAW)	3 - 15	7.80 ±2.83	7.0 [3.0]	86 (35.7)	114 (47.3)	41 (17.0)
Total scores of QWL	34 - 116	64.27 ±14.08	65.0 [18.0]	66 (27.4)	168 (69.7)	7 (2.9)
Overall (in response with a single question)	1 - 5	2.57 ±1.12	3.0 [1.0]	55 (22.8)	139 (57.7)	47 (19.5)
**Burnout subscales (*n* = 241)**	**Min-Max**	**Mean ±SD**	**Median [IQR]**	**Burnout levels, n (%)**		
				**Low**	**Moderate**	**High**
Emotional exhaustion (EE)	0 - 54	36.15 ±14.31	40.0 [18.0]	32 (13.3)	18 (7.5)	191 (79.3)
Depersonalization (DP)	0 - 30	14.11 ±7.73	15.0 [12.0]	56 (23.2)	48 (19.9)	137 (56.8)
Personal accomplishment (PA)	0 - 48	27.28 ±10.50	29.0 [14.0]	137 (56.8)	76 (31.5)	28 (11.6)

Emotional exhaustion is classified as low ≤ 18 scores, moderate = 19-26 scores, and high ≥ 27 scores; Depersonalization is classified as low ≤ 5 scores, moderate = 6-9 scores, and high ≥ 10 scores; Personal accomplishment is classified as low ≤ 33 scores, moderate = 34-39 scores, and high ≥ 40 scores.
