# Supplementary material for: Burnout and Quality of Work Life among Physicians during Internships in Public Hospitals in Thailand
Source: Behav Sci (Basel). 2024 Apr 25;14(5):361. doi: 10.3390/bs14050361 (PMC11117651; doi:10.3390/bs14050361)
Supplement: Supplementary file 1 [file behavsci-14-00361-s001.zip › Supplementary Table S4.pdf]

Table S4. Frequency and percentage of quality of work-life levels among intern physicians.

Item	Questions (*n* = 241)	***n* (%)** Strongly disagree	Disagree	Neutral	Agree	Strongly agree
JCS1	I have a clear set of goals and purposes in doing my job.	26 (10.8)	25 (10.4)	73 (30.3)	85 (35.3)	32 (13.3)
CAW1	I feel able to voice opinions and influence changes in my area of work.	47 (19.5)	62 (25.7)	69 (28.6)	47 (19.5)	16 (6.6)
JCS2	I have the opportunity to use my abilities at work.	18 (7.5)	22 (9.1)	77 (32.0)	99 (41.1)	25 (10.4)
HWI1	My employer provides adequate facilities and flexibility for me to fit work around my family life.	44 (18.3)	67 (27.8)	83 (34.4)	39 (16.2)	8 (3.3)
HWI2	My current working hours/ patterns suit my personal circumstances.	67 (27.8)	100 (41.5)	51 (21.2)	20 (8.3)	3 (1.2)
SAW1	I often feel under pressure at work. ≠	53 (22.0)	114 (47.3)	35 (14.5)	17 (7.1)	22 (9.1)
JCS3	When I have done a good job, it is acknowledged by my line manager.	46 (19.1)	20 (8.3)	88 (36.5)	71 (29.5)	16 (6.6)
GWB1	Recently, I have been feeling unhappy and depressed. ≠	38 (15.8)	88 (36.5)	52 (21.6)	31 (12.9)	32 (13.3)
JCS4	I am encouraged to develop new skills.	30 (12.4)	44 (18.3)	91 (37.8)	64 (26.6)	12 (5.0)
CAW2	I am involved in decisions that affect me in my own area of work.	44 (18.3)	42 (17.4)	80 (33.2)	65 (27.0)	10 (4.1)
WCS1	My employer provides me with what I need to do my job effectively.	63 (26.1)	58 (24.1)	88 (36.5)	29 (12.0)	3 (1.2)
GWB2	In most ways, my life is close to ideal.	87 (36.1)	87 (36.1)	54 (22.4)	11 (4.6)	2 (0.8)
WCS2	I work in a safe environment.	36 (14.9)	59 (24.5)	77 (32.0)	58 (24.1)	11 (4.6)
GWB3	Generally, things work out well for me.	31 (12.9)	47 (19.5)	107 (44.4)	52 (21.6)	4 (1.7)
JCS5	I am satisfied with the career opportunities available for me here.	41 (17.0)	38 (15.8)	90 (37.3)	59 (24.5)	13 (5.4)
JCS6	I am satisfied with the training I received in order to perform my present job.	38 (15.8)	29 (12.0)	88 (36.5)	69 (28.6)	17 (7.1)
GWB4	Recently, I have been feeling reasonably happy all things considered.	44 (18.3)	29 (12.0)	106 (44.0)	56 (23.2)	6 (2.5)
SAW2	I have an unachievable deadline. ≠	21 (8.7)	116 (48.1)	38 (15.8)	29 (12.0)	37 (15.4)
HWI4	I am able to achieve a healthy balance between my work and home life.	53 (22.0)	76 (31.5)	68 (28.2)	37 (15.4)	7 (2.9)
EET1	I am proud to tell others that I am part of this organization.	44 (18.3)	53 (22.0)	85 (35.3)	49 (20.3)	10 (4.1)
EET2	I would recommend this organization as a good one to work for.	75 (31.1)	54 (22.4)	66 (27.4)	36 (14.9)	10 (4.1)
SAW3	I am pressured to work long hours. ≠	47 (19.5)	85 (35.3)	45 (18.7)	23 (9.5)	41 (17.0)
CAW4	I have sufficient opportunities to question managers about the changes at work.	78 (32.4)	72 (29.9)	59 (24.5)	25 (10.4)	7 (2.9)
WCS3	I am happy with the physical environment where I usually work.	50 (20.7)	51 (21.2)	88 (36.5)	45 (18.7)	7 (2.9)

Item	Questions (n = 241)	n (%)				
		**Strongly disagree**	**Disagree**	**Neutral**	**Agree**	**Strongly agree**
OVL	I am satisfied with the overall quality of my working life.	55 (22.8)	50 (22.8)	89 (36.9)	38 (15.8)	9 (3.7)

≠ Negative question; n = Number of responses in each question. Abbreviations: EET, Employee engagement; CAW, Control at work; HWI, Homework interface; GWB, General well-being; JCS, Job and career satisfaction; WCS, Working conditions; SAW, Stress at work; OVL, Overall (in response with a single question).
